# Supplementary material for: Thirteen dubious ways to detect conserved structural RNAs
Source: IUBMB Life. Author manuscript; Available in PMC 2024 Jul 10. (PMC11234323; doi:10.1002/iub.2694)
Supplement: supplementary_material [file NIHMS1907110-supplement-supplementary_material.gz › supplemental_material/rscape_v2.0.0.k.tar.gz!/rscape_v2.0.0.k/logo/R-scape_logo_final_.pdf]

R-scape
